# Exploring differentially expressed genes of *Staphylococcus* *aureus* exposed to human tonsillar cells using RNA sequencing

**DOI:** 10.1186/s12866-023-02919-5

**Published:** 2023-07-12

**Authors:** Srijana Bastakoti, Clement Ajayi, Kjersti Julin, Mona Johannessen, Anne-Merethe Hanssen

**Affiliations:** 1 Department of Medical Biology, Host-Microbe Interaction (HMI) research group, UiT – The Arctic University of Norway, Tromsø, Norway; 2 Center for Research and Education, University Hospital of North Norway (UNN), Tromsø, Norway

**Keywords:** *Staphylococcus aureus*, Human tonsil epithelial cells, Throat colonization, Transcriptomics, RNA sequencing

## Abstract

**Background:**

The nose and the throat are the most predominant colonizing sites of *Staphylococcus aureus*, and colonization is a risk factor for infection. Nasal colonization is well described; however, we have limited knowledge about *S. aureus* throat colonization. The main objective of this study was to explore differentially expressed genes (DEGs) in *S. aureus* throat isolate TR145 exposed to human tonsil epithelial cells (HTEpiC) by using RNA sequencing (RNA-seq) and pathway analysis. DEGs in *S. aureus* at 1 or 3 hours (h) interaction with its host were explored.

**Results:**

*S. aureus* was co-cultured in absence and presence of tonsillar cells at 1 or 3 h. Over the 3 h time frame, the bacteria multiplied, but still caused only minor cytotoxicity. Upon exposure to tonsillar cell line, *S. aureus* changed its transcriptomic profile. A total of 508 DEGs were identified including unique (1 h, 160 DEGs and 3 h, 78 DEGs) and commonly shared genes (1 and 3 h, 270 DEGs). Among the DEGs, were genes encoding proteins involved in adhesion and immune evasion, as well as iron acquisition and transport. Reverse transcription qPCR was done on selected genes, and the results correlated with the RNA-seq data.

**Conclusion:**

We have shown the suitability of using HTEpiC as an in vitro model for investigating key determinants in *S. aureus* during co-incubation with host cells. Several DEGs were unique after 1 or 3 h exposure to host cells, while others were commonly expressed at both time points. As their expression is induced upon meeting with the host, they might be explored further for future targets for intervention to prevent either colonization or infection in the throat.

**Supplementary Information:**

The online version contains supplementary material available at 10.1186/s12866-023-02919-5.

## Background

*Staphylococcus aureus* is an opportunistic pathogen that can cause life-threatening diseases such as endocarditis, osteomyelitis, pneumonia, and bacteremia [1]. Besides being a human pathogen, *S. aureus* asymptomatically colonizes 20–30% of a healthy adult population [2, 3]. Colonization is a risk factor for infection, as the colonizing strain is responsible for approximately 80% of *S. aureus* infections within its host [1, 4].

The predominant and frequent colonizing sites for *S. aureus* are the vestibulum nasi (anterior nares) followed by skin, perineum, and pharynx [3, 5]. *S. aureus* encodes various adhesive proteins, including microbial surface components recognizing adhesive matrix molecules (MSCRAMMs), that are involved in adhesion to the host cells [6]. Several of these are expressed during nasal colonization [7]. During colonization, *S. aureus* not only adhere to cell surfaces but is also intracellularly [8], which may protect the bacteria against antibiotic treatment [9]. In addition, the pathogen also expresses proteins to overcome host immune defence mechanisms and retrieve iron from the host [7]. Although the nares are considered the primary site of *S. aureus* colonization [5, 8], pharyngeal *S. aureus* carriage has also been equally or more commonly observed [4, 10–12]. Both methicillin-sensitive (MSSA) and methicillin-resistant *S. aureus* (MRSA) can persistently colonize the throat of healthy people for years [13, 14], and 32.1% of colonizing MRSA strains have been exclusively isolated from throat carriers [13].

Prevention and elimination of the *S. aureus* colonization carrier state may contribute to reduce the *S. aureus* infection burden and prevent the spread of MRSA [2, 15]. Mupirocin is widely used for the de-colonization of *S. aureus* in the nasal cavity. It interferes with the synthesis of bacterial proteins by reversibly binding to bacterial isoleucyl-tRNA [16]. However, *S. aureus* colonization frequently reoccurs after mupirocin treatment [17]. The ability of *S. aureus* to colonize the throat region makes it difficult to be reached by mupirocin treatment, and throat colonization has been shown to be linked to reduction in the eradication efficacy [18]. Hence, new antimicrobial compounds for *S. aureus* de-colonization, especially in throat, are urgently needed.

This study aimed to identify key determinants differentially expressed by *S. aureus* in presence of primary human tonsil epithelial cells using RNA sequencing (RNA-seq) and pathway analysis.

## Results

### *S. aureus* multiply in presence and absence of tonsillar cells

*S. aureus* TR145, isolated from a healthy adult that was exclusively throat colonized, was chosen as a representative throat strain in our study. To determine the effect of exposure to tonsillar cells on the growth of *S. aureus* TR145, bacteria grown to log phase OD_600nm_ = 0.8–1.2 were diluted to OD_600nm_ 0.4 and seeded into wells with or without human tonsil epithelial cells at a MOI of 5. The CFU of the *S. aureus* inoculum used to infect either host media or the host cells were ~ 1.4 × 10^6^ CFU/ml, corresponding to 6.2 log10 CFU/ml.

**Fig. 1:**
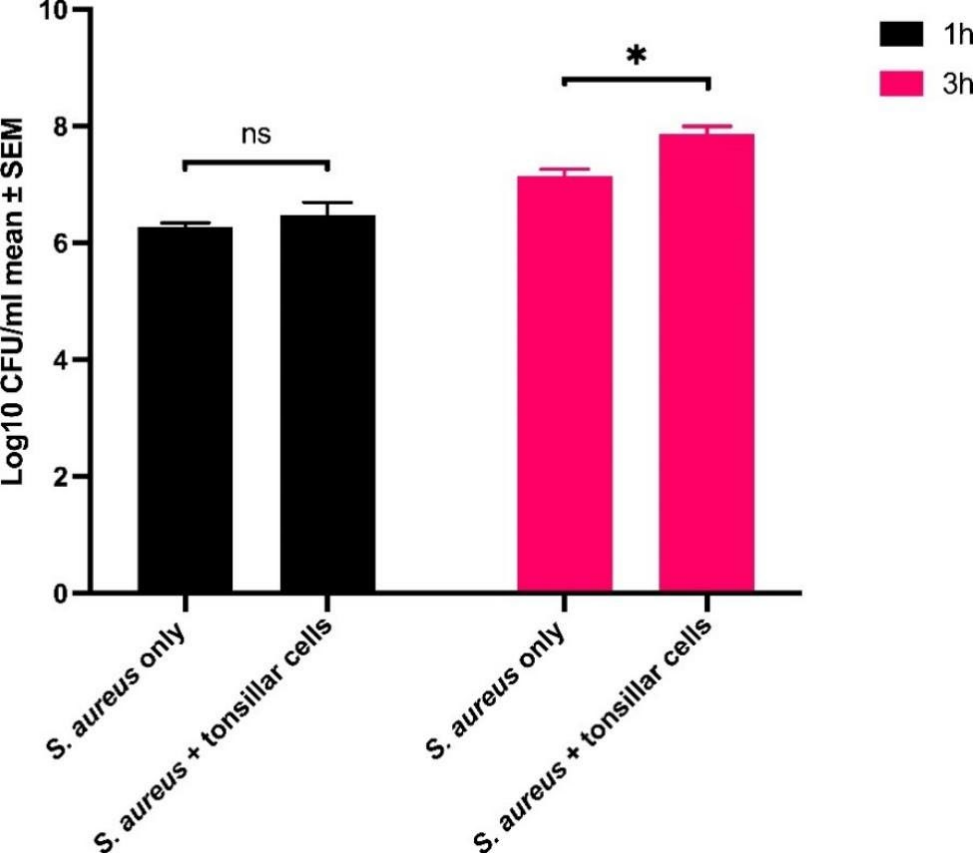
The growth of *S. aureus* TR145 in absence or presence of human tonsillar epithelial cells. The results are presented as log10 CFU/ml from three independent experiments. The different colors show the *S. aureus* grown at different time points (1 and 3 h) either alone or together with tonsillar cells. Paired-t test was performed separately for two different time points to compare growth of *S. aureus* only with *S. aureus* mixed with tonsillar cells. At 1 h of exposure, there was no significant (ns) difference between *S. aureus* alone and *S. aureus* with tonsillar cells. Whereas, at 3 h of exposure, significantly difference (p < 0.005, *) was observed

There was no significant difference in the recoverable CFU between *S*. *aureus* TR145 exposed to tonsillar cells compared to those grown without the host cells at 1 h; however, there was almost 1 log10 difference in bacterial growth when grown in presence of host cells for 3 h (Fig. 1).

**Fig. 2:**
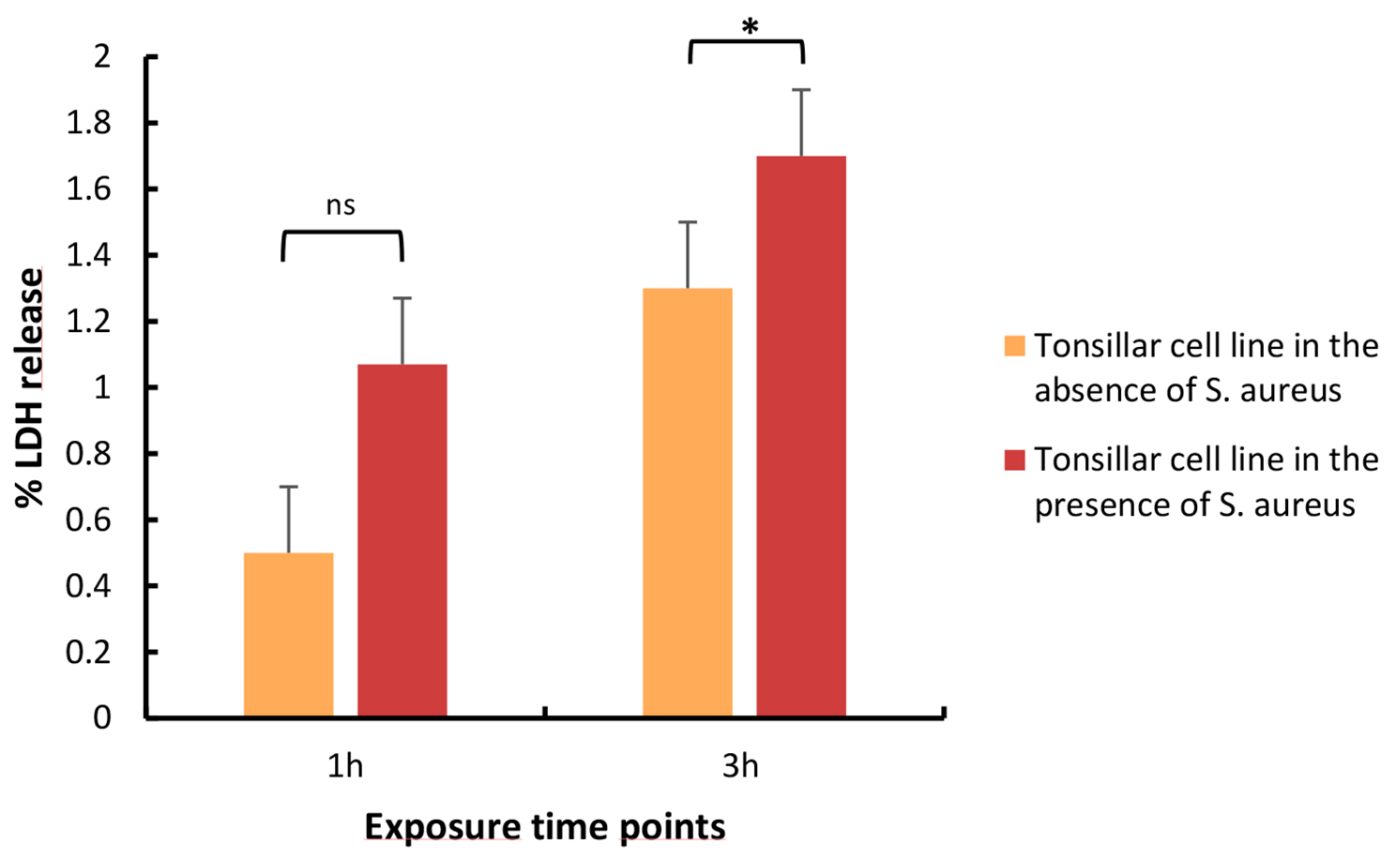
*S. aureus* shows minor cytotoxicity to the tonsillar cell line. LDH release from tonsillar cells into the supernatant was measured following exposure with/without *S. aureus* for 1 and 3 h. The orange bar represents the percentage (%) of LDH released by the tonsillar cell line in the absence of *S. aureus* (negative control) whereas red bar presents the LDH % release by the tonsillar cell line in the presence of *S. aureus*. The bacterial cytotoxicity was calculated as a percentage of maximum LDH release control (positive control). The results are based on three independent experiments

As we aimed to analyze bacterial transcriptome in presence of human tonsil epithelial cells, we next evaluated the host cell viability after exposure to *S. aureus*. The bacterial effect on host cell viability was evaluated by measuring the lactase dehydrogenase (LDH) release by the tonsillar cells in presence or absence of *S. aureus*. As shown in Fig. 2, there was no significant difference in the LDH release from host cells after 1 h growth in absence or presence of *S. aureus*. The LDH release increased slightly after 3 h growth in absence (1.3%) or presence of *S. aureus* (1.7%).

Thus, *S. aureus* can multiply in absence and presence of the host cells, causing only minor cytotoxicity.

### RNA sequencing and analysis of RNA-seq data

Total RNA from *S. aureus* exposed to growth medium or host cells were isolated and used for RNA-seq library preparation. The quantity of RNA measured by Nanodrop1000 spectrophotometer detected RNA concentrations ranging from 1.5 ng/µl − 80.6 ng/µl in the samples of *S. aureus* in absence of host cells and 23.2 ng/µl – 876 ng/µl in samples of *S. aureus* exposed to host (Additional file 1, Table S1). For most of the RNA samples, the RNA quality and integrity analyzed by Agilent 2100 Bioanalyzer detected an RNA integrity number (RIN) score of > 7.0.

RNA-seq generated 34.2–53.6 million total reads per library. Only sequences with quality score Q ≥ 20 were retained in the dataset. The reads remaining after trimming and filtering of low-quality bases and adaptor contaminants were 33.3–53.5 million reads per library (Additional file 2, Table S2). The filtered reads were then mapped with reference genome *S. aureus* TR145 (SAMEA112465883). All the RNA samples for the 3 h time point showed more than 55% of RNA reads uniquely mapped with its reference genome. In comparison, at 1 h of exposure condition, most of the samples showed less than 20% of mapping efficacy although one sample (C1h_Rep1) showed 93% of mapping efficacy (Additional file 2, Table S2). In summary, high-quality RNA was retrieved and used for RNA-seq, and only quality reads mapped against *S. aureus* TR145 were accessed for RNA-seq data analysis.

### Data normalization and visualization of sample variance

**Fig. 3:**
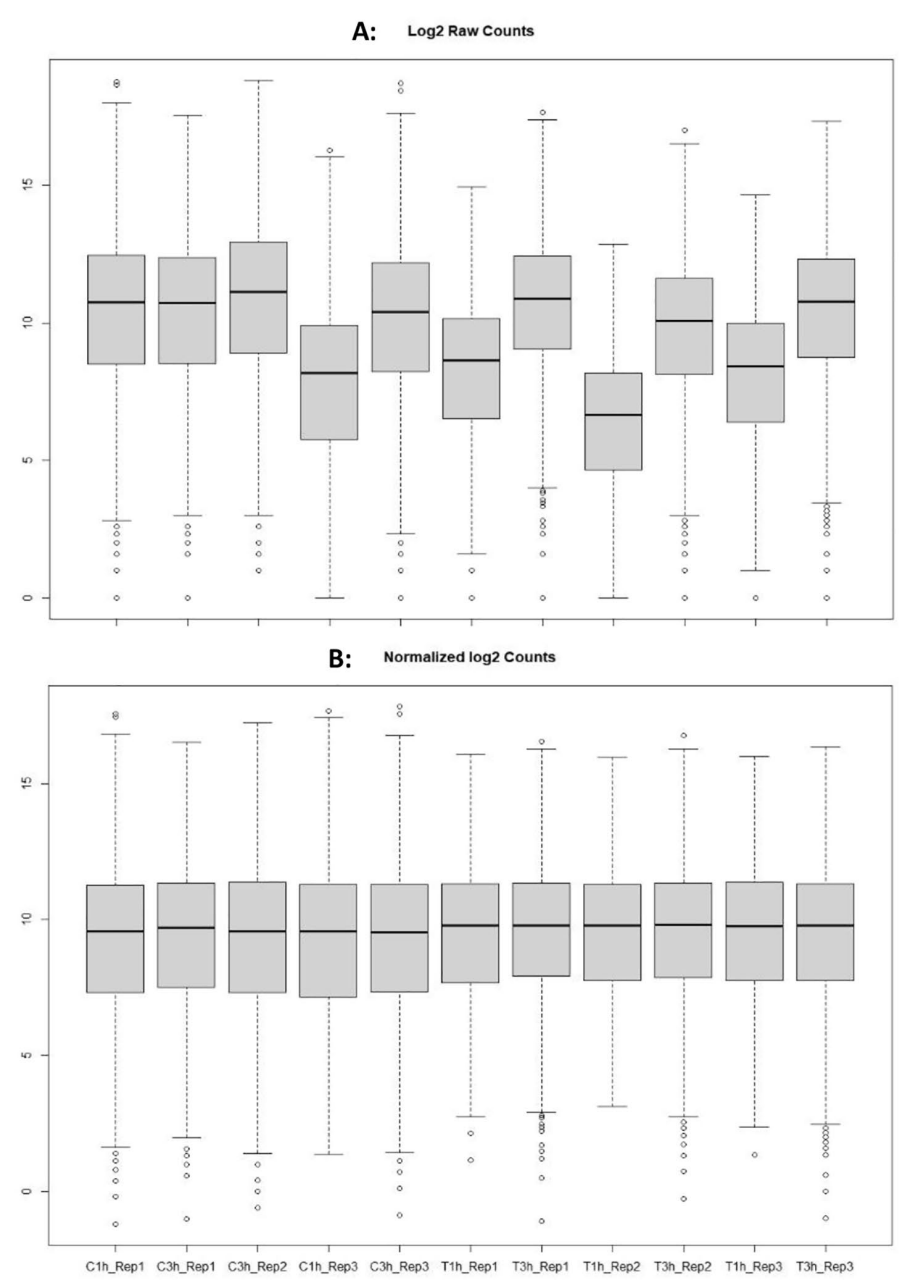
Quality control of RNA-seq transcriptomics data presented in box plot before differentially expressed gene analysis in *S. aureus* exposed to tonsillar cells. The three biological replicates consist of *S. aureus* only 3 h (C3h), *S. aureus* exposed to tonsillar cells 1 h (T1h) and *S. aureus* exposed to tonsillar cells 3 h (T3h). There were two replicates for *S. aureus* only 1 h (C1h), as one sample was lost under preparation ahead of RNA-seq. The DESeq2 uses the median of ratio method for normalizing the counts and is depicted in log2 fold change. **A**: The distribution of raw counts before normalization. **B**: The counts after performing DESeq2 normalization

The normalization of read counts was used for gene count comparisons between *S. aureus* alone (1 h and 3 h) or exposed growth for 1 h and 3 h. After normalization, both sequencing depth and RNA composition were corrected, including the difference in biological replicates (Fig. 3).

**Fig. 4:**
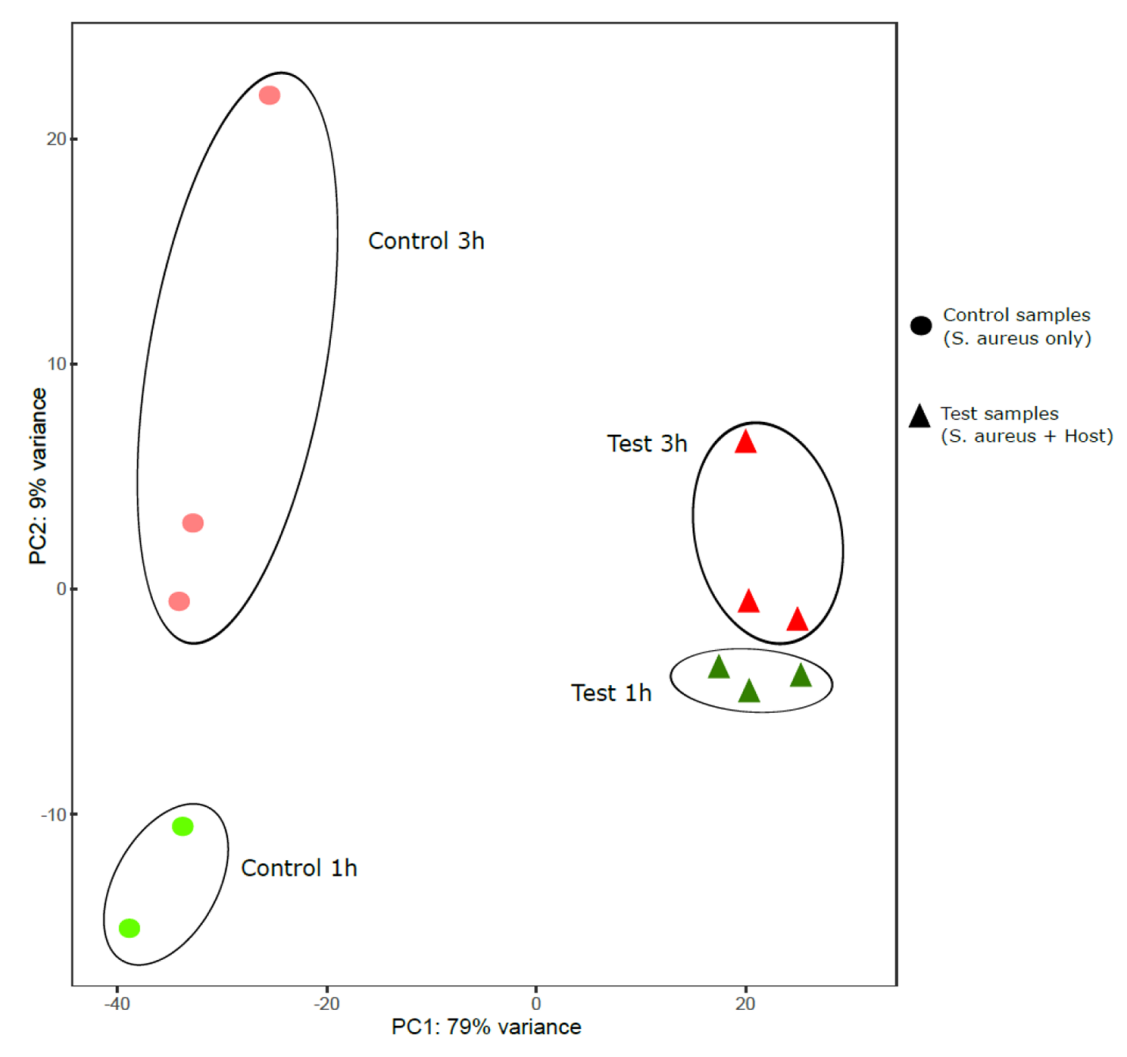
Principal component analysis (PCA) analysis of RNA-seq data to visualize sample to sample variation. The PCA plot depicted clear clustering control (*S. aureus* only, shape- circle) versus test samples (*S. aureus* + tonsillar cells, shape-triangle). The test samples, *S. aureus* after 1 h (n = 3; dark green) and 3 h (n = 3; dark red) exposure to tonsillar cells cluster separately to that of control samples (n = 2, light green) at 1 h and (n = 3, light red) at 3 h. The first two components PC1 and PC2 explained 79% and 9% of the variability in the expression data, respectively

The principal component analysis (PCA) plot (Fig. 4) was used to visualize the sample variation between *S. aureus* grown in presence or absence of host cells. The results showed a clear clustering of the three biological replicates of *S. aureus* harvested at 1 or 3 h in presence or absence of tonsillar cells (Fig. 4).

These analyses indicate that all the variations which might have occurred due to biological replicates and sequencing depth/cycle have been normalized, and clear clustering of different sample groups are visualized, making it ready for DEGs analysis.

### Differentially expressed genes (DEGs) in ***S. aureus*** in presence and absence of tonsillar cells

**Fig. 5:**
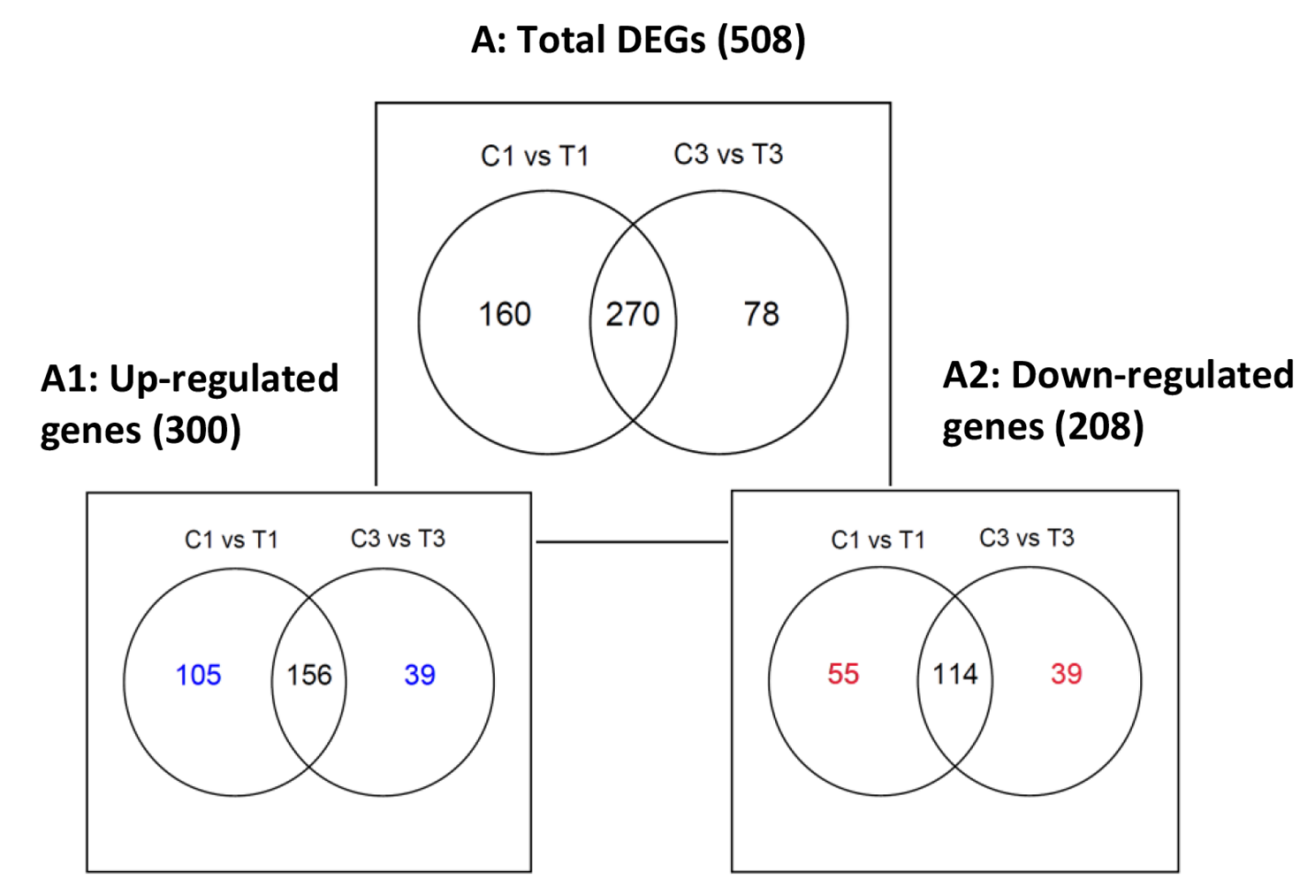
Venn diagram of differentially expressed genes (DEGs) in *S aureus* in the presence of tonsillar cells identified by RNA-seq. A: Represents the total DEGs (508), thereof uniquely expressed at 1 h (160) or 3 h (78) exposure to host cells or expressed at both time points (270). **A1**: Representation of 300 up-regulated genes, uniquely expressed 105 and 39 genes identified at 1 and 3 h, respectively. **A2**: Representation of 208 down-regulated genes, uniquely expressed 55 and 39 genes identified at 1 and 3 h, respectively

To investigate the changes in *S. aureus* gene expression after exposure to tonsillar cells, computational comparisons analysis of the DEGs from *S. aureus* exposed or not exposed to tonsillar cells was performed. The DESeq2 analysis of the HTseq generated gene count files revealed a total of 508 significant DEGs with adjacent p value (padj) < 0.05 and log2fold change (lfc) ≥ |2|) from *S. aureus* TR145 exposed to tonsillar cells at 1 and 3 h compared to those grown in the absence of tonsillar cells at these time points (Fig. 5).

Two hundred and seventy DEGs were expressed both after 1 and 3 h exposure to host cells, while 160 and 78 DEGs were uniquely expressed at 1 and 3 h, respectively (Fig. 5A). Three hundred DEGs, (59%) were significantly upregulated in presence of host cells, whereof 156 were commonly expressed, while 105 and 39 DEGs were uniquely expressed at 1 and 3 h, respectively (Fig. 5A1). A total of 208 DEGs (41%) were significantly downregulated, whereof 114 were commonly expressed, and 55 and 39 were uniquely expressed at 1 and 3 h respectively (Fig. 5A2).

For *S. aureus* exposed to host cells for 1 h, 430 genes (160 + 270 Fig. 5A) were significantly differentially expressed, and among these 310 were pre-annotated. Of these, 105 genes were uniquely expressed at 1 h, and their identity and lfc expression are presented in Additional file 3, Table S3 including commonly shared DEGs (205 pre-annotated). After 3 h exposure to host cells, 348 (270 + 78, Fig. 5A) significant DEGs were found, and among these, 53 pre-annotated genes were uniquely expressed after 3 h. Their identity and lfc are presented in Additional file 4, Table S4 including commonly expressed genes. A complete list of DESeq2, without any threshold cutoff is presented in additional file 5, Table S5. In summary, the *S. aureus* transcriptome changed upon exposure to tonsillar cell line. Some transcripts were unique for the tested time points, while others were expressed at both time points.

### Enriched gene ontology (GO) terms in ***S. aureus***

**Fig. 6:**
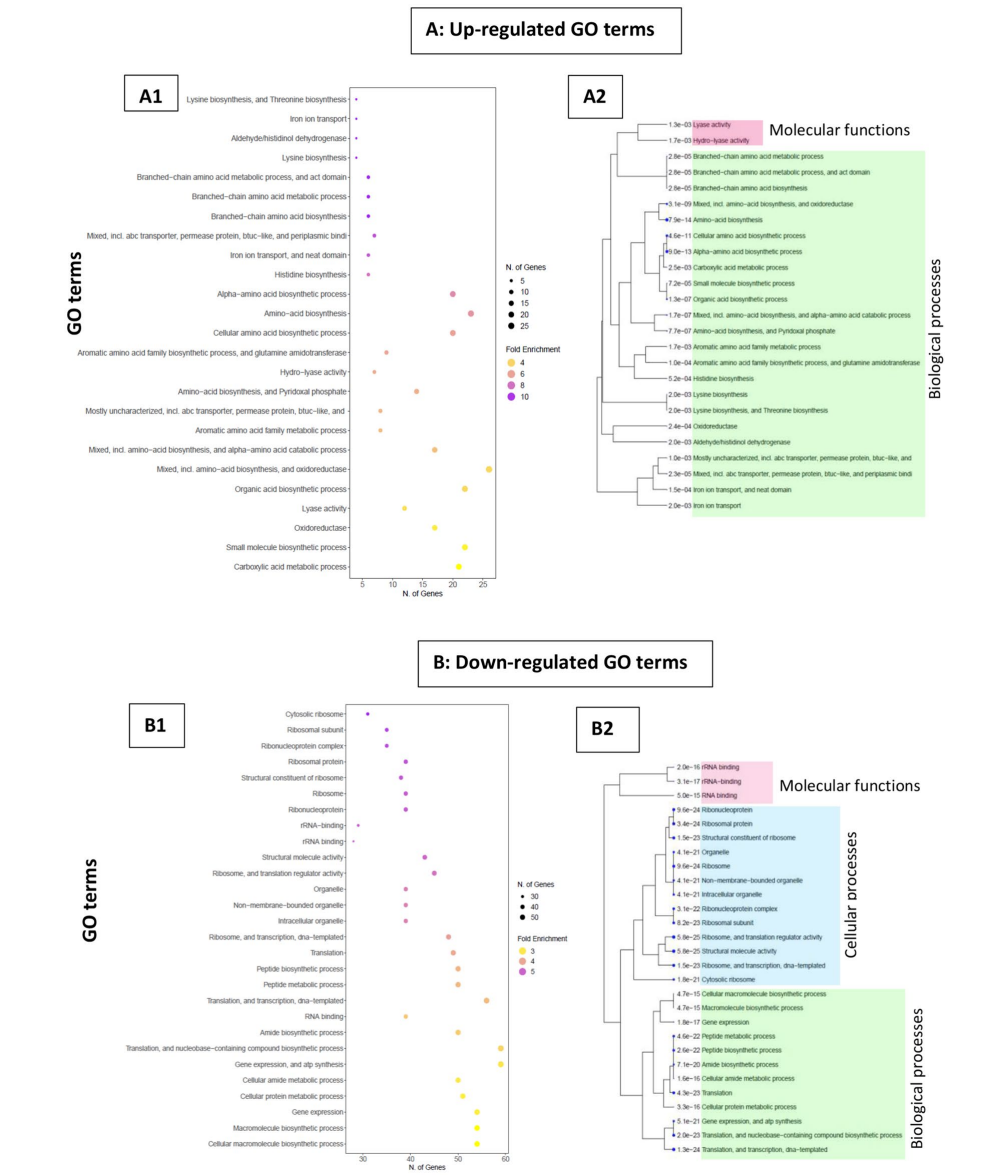
Gene Ontology (GO) analysis of identified differentially expressed genes (DEGs) in *S. aureus* after 1 and 3 h of exposure with tonsillar cells. **A1** and **B1** represent the GO-enriched scatter plots, identified within top 30, from upregulated and downregulated genes The size of the dot represents the number of DEGs, and the color represents level of fold enrichment of the identified GO terms. **A2** and **B2** represent the hierarchical clustering tree diagram from GO analysis, showing different clades of GO-enriched terms divided into biological processes, cellular processes, or molecular components

Sets of all upregulated (300) and downregulated (208) genes derived from RNA-seq data analysis were separately applied in gene ontology (GO) enrichment analysis to identify enriched pathways involved during *S. aureus* exposure to tonsillar cells. The most enriched upregulated and downregulated GO terms identified are presented in Fig. 6A and B, respectively. Enriched GO terms were first filtered based on false discovery rate (FDR) cutoff (< 0.05), then the significant pathways were selected by FDR and sorted by Fold Enrichment.

The upregulated genes identified significantly (FDR < 0.05, fold enrichment ≤ 10) enriched pathways involved either in biological process or molecular function (Fig. 6A1). About 10 genes (FDR < 0.05, 8 ≥ fold enrichment ≤ 10) were involved in the highly enriched biological processes such as amino acid biosynthesis process, iron ion transport, neat domain, etc. (Fig. 6A1; small circles in purple). Only two GO terms, lyase and hydro-lyase activity, were involved in molecular function (Fig. 6A2).

GO term analysis from all downregulated genes revealed several enriched GO terms involved during cellular process followed by biological and molecular activity (Fig. 6B1). About 45 genes (FDR < 0.05, 3 > fold enrichment ≤ 5) belong to the topmost enriched downregulated GO terms including cytosolic ribosome, ribosomal subunit/protein, rRNA binding, and organelle (Fig. 6B1; circles in dark to light purple from the left). The correlation among the significant pathways for the up and downregulated genes are presented in hierarchical clustering tree diagram (Fig. 6A2, 6B2).

Taken together, GO analysis performed for DEGs were found to be highly involved in biological processes followed by cellular and in molecular pathways.

**Fig. 7:**
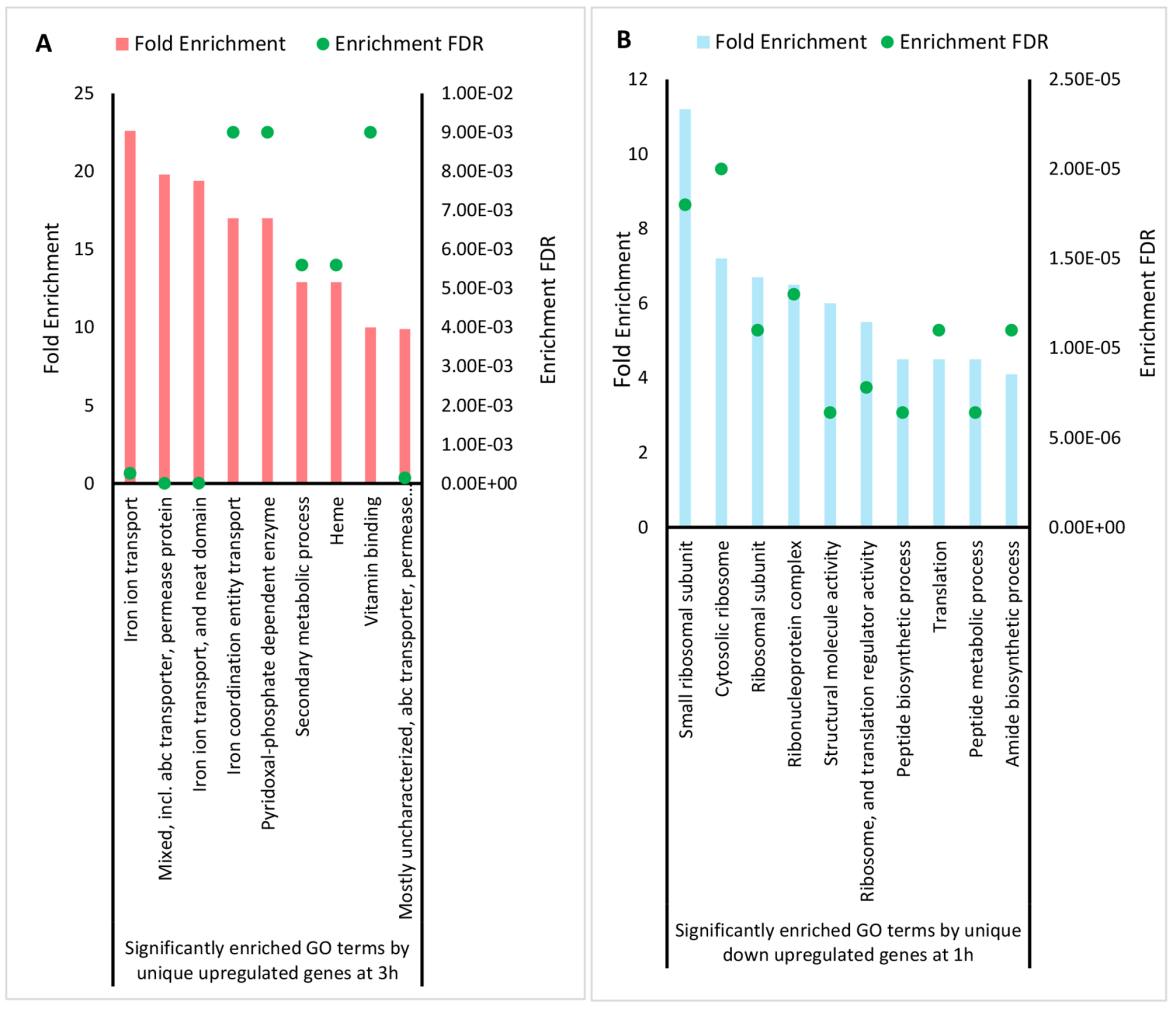
Different significantly enriched top 10 GO terms analyzed from unique differentially expressed genes at 1 and 3 h of exposure with tonsillar cells. Horizontal line represents the different significant GO terms identified by GO analysis, left vertical represents the fold enrichment value, and right vertical line with enrichment false discovery rate (FDR). **A)** The uniquely upregulated genes at 3 h involved in top 9 GO terms. All red bars indicate the significant GO terms. No significantly enriched GO terms were identified from unique down regulated genes at 3 h in the GO analysis with FDR < 0.05. **B)** The uniquely downregulated at 1 h involved in top 10 GO terms. All blue bars indicate the significant GO terms. No significantly enriched GO terms were identified from unique up regulated genes at 1 h in the GO analysis with FDR < 0.05

Further, to visualize the major pathway differences on uniquely regulated DEGs after 1 and 3 h exposure to tonsillar cells, the 10 topmost significantly enriched GO terms were analyzed. It resulted in 9 enriched GO terms from 3 h, and 10 enriched GO terms from 1 h (Fig. 7). One of the highly enriched pathways (FDR < 0.05, fold enrichment 35) detected from upregulated genes at 3 h was the iron ion transport (Fig. 7A). In contrast, no significantly enriched pathways were found at 1 h among highly upregulated genes using GO analysis with FDR < 0.05. Uniquely downregulated genes at 1 h revealed significantly enriched pathways (FDR < 0.05, fold enrichment ≤ 10) such as small ribosomal subunits, translation, and biosynthetic process (Fig. 7B). No significant enriched pathways were found among uniquely downregulated genes at 3 h using GO analysis with FDR < 0.05.

Overall, this indicates that the bacteria downregulate transcription of genes encoding proteins involved in translational process after 1 h exposure to host. After 3 h exposure, the bacteria might be faced with competition for ions, and upregulate transcription of genes encoding proteins involved in iron acquisition and transport.

The uniquely upregulated genes at 3 h were further explored to identify several other significantly enriched biological processes involved during *S. aureus* exposure to tonsillar cells. The 20 topmost biologically enriched pathways analysis resulted in 16 significantly enriched GO terms (Additional file 6, Figure S1).

Most of the biologically enriched pathways were involved in biological adhesion, biosynthesis in addition to iron acquisition and transport. DEGs associated with iron acquisition and transport and biological adhesion are listed in Table 1.

**Table 1:** Top biological processes enriched by uniquely upregulated genes at 3 h identified from GO analysis. Genes were significantly involved (FDR < 0.05) in the respective pathways detected by GO enrichment analysis

Fold Enrichment	GO terms	Genes
36	Iron import into cell	*isdI, isdF*
27	Iron coordination entity transport	*isdC, isdE, isdF*
24	Iron ion transport	*isdI, isdC, isdE, isdF*
24	Cellular metal ion homeostasis	*isdI, isdF*
24	Heme transport	*isdC, isdE*
24	Establishment of localization in cell	*isdI, isdF*
24	Iron ion homeostasis	*isdI, isdF*
18	Cell and biological adhesion	*sdrC, icaA*
18	Cellular cation homeostasis	*isdI, isdF*
8	Transition metal ion transport	*isdI, isdC, isdE, isdF*
6	Alpha-amino acid biosynthetic process	*sbnA, argH, argG, trpF, trpB*

### Expression of ***S. aureus*** survival factors when exposed to tonsillar cells

**Fig. 8:**
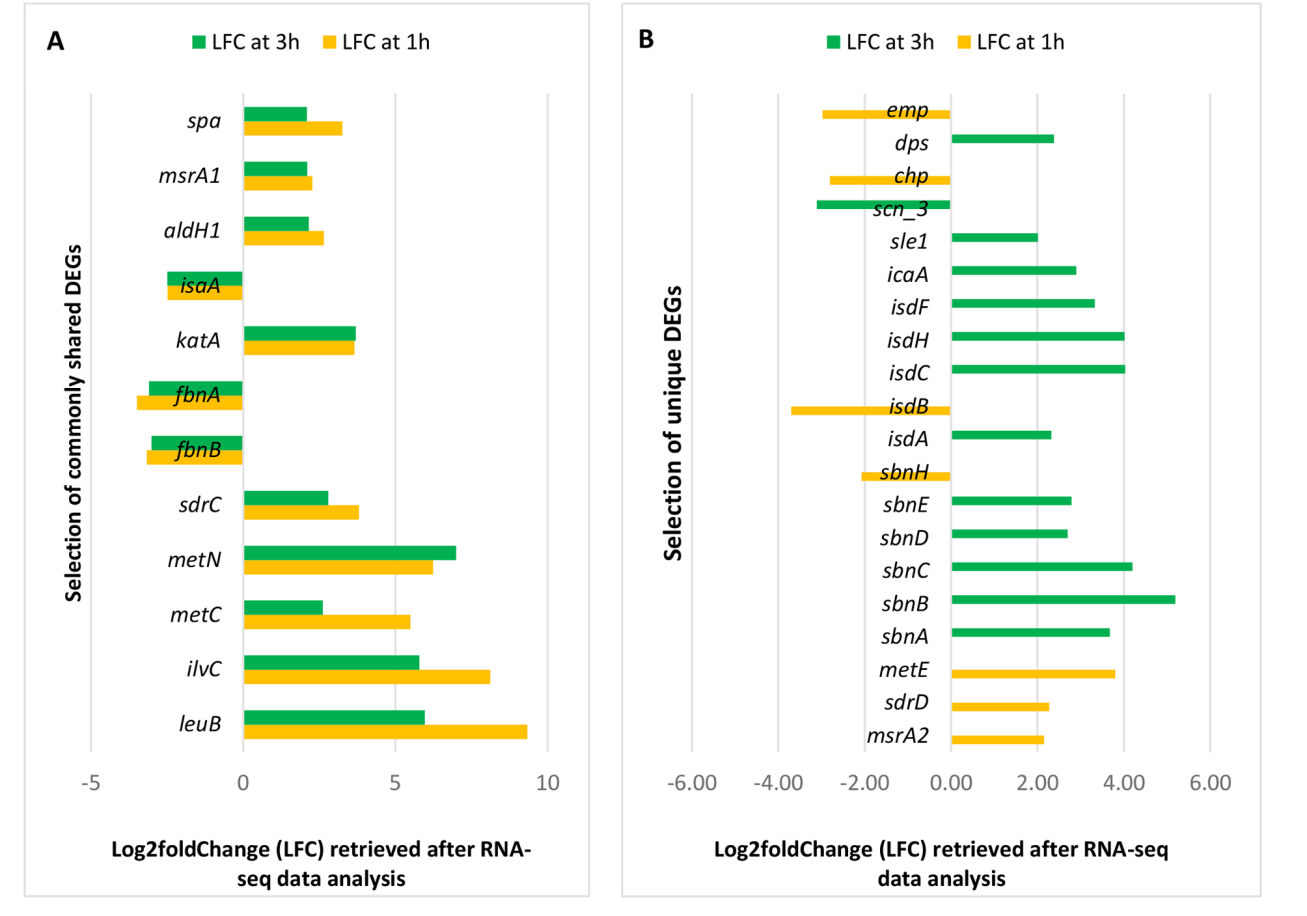
Selected common and unique DEGs in *S. aureus* exposed to tonsillar cells at 1 and 3 h identified from RNA-seq. Log2fold change retrieved after RNA-seq analysis. **(A)** DEGs detected both at 1 and 3 h after exposure to host cells. **(B)** DEGs uniquely expressed either at 1 or 3 h after exposure to host cells

The genes presented in Fig. 8, were selected with respect to gene expression level, and functions involved in adhesion, iron acquisition/transport and amino acid synthesis, as these are relevant when the bacteria meet the host, identified from GO enrichment analysis. Among the commonly shared upregulated genes were *leuB, ilvC, metC, metN, sdrC, katA, aldH1, msrA1* and *spa.* Some of the downregulated genes were fibronectin binding genes *fnbA* and *fnbB* as well as staphylococcal antigen A (*isaA*) (Fig. 8A).

The upregulated genes responsible for iron acquisition and transport namely *isdACEFHI* and *sbn*ABCDE were only expressed at 3 h exposure to host cells. Some of the genes such as *dps, sle1* and *icaA* were also uniquely upregulated after 3 h of exposure to tonsillar cells. After 1 h exposure, the genes *metE, sdrD* and *msrA2* were upregulated while *emp, chp* and *sbnH* were downregulated (Fig. 8B).

In summary, some of the DEGs were present both after 1 h and 3 h exposure to host, which indicates a more constant need in making proteins that help in e.g., methionine biosynthesis and destruction of hydrogen peroxide (katA). Similarly, some of the unique DEGs at 1 h were found to be involved in cell division and methionine synthesis, whereas at 3 h of exposure, *S. aureus* mostly expressed genes having a role in iron acquisition, iron hemostasis including cell attachment and stress response. These results demonstrate *S. aureus* ability to adapt to the changing environment that might occur over time.

### RT-qPCR confirmation

To verify the DEGs obtained from the RNA-seq datasets of *S. aureus* during exposure to tonsillar cells, reverse transcription qPCR experiments were performed for six genes (*ilvC, metI, metE, icaA, emp*, and *rpsT*). For all the six genes tested, the RT-qPCR data showed a general correlation with RNA-seq data in log2fold changes, where three of them were significant (Table 2). Overall, the correlation between RNA-seq and RT-qPCR is good, validating the RNA-seq data.

**Table 2:** RNA-seq data validation by RT-qPCR using six selected genes (*ilvC*, *metI*, *metE*, *icaA*, *emp*, and *rpsT*). RNA-seq values represent log_2_ fold change of transcripts inferred by bioinformatics prediction. RT-qPCR values represent the mean log_2_ fold change in transcripts. The value represents the log2 of the relative fold change between control (*S. aureus* only) and test (*S. aureus* with host). For both the analysis, log_2_ fold change and adjusted p-value is presented. The p-value of less than 0.05 was significant

	Log2fold change		Adjusted P value	
**Gene Symbol**	**RNA seq**	**RT qPCR**	**RNA-seq**	**qRT-PCR**
*ilvc*	8.18	35	0.0000	0.0280
*metI*	2.95	2	0.0000	0.3351
*metE*	3.8	5	0.0000	0.1472
*icaA*	2.95	6	0.0009	0.0043
*emp*	-2.97	-1	0.0004	0.3831
*rpst*	-2.23	0.7	0.0000	0.0050

## Discussion

In this study, we used the human tonsillar cell line (HTEpiC) to identify possible *S. aureus* determinants involved during *S. aureus* throat colonization. The suitability of using HTEpiC as an in vitro model for investigating the key determinants in *S. aureus* exposure to human tonsillar cells has not been previously verified, making our study the first to investigate this interaction. We observed that after 1 or 3 h of *S. aureus* presence, the host cells remained viable, as indicated by the negligible levels of LDH released by the host cells. This finding suggests that the HTEpiC cell line is suitable for studying the interaction between *S. aureus* and human tonsillar cells without compromising the viability of the host cells.

Numerous transcriptomics studies have investigated the transcriptomics profiling of *S. aureus* during colonization of the nasal and vaginal regions, as well as during infections of the lung and skin [19–22]. However, there is limited knowledge about *S. aureus* transcriptome in throat colonization. To the best of our knowledge, the present study is the first to investigate the unique determinants expressed by *S. aureus* when exposed to tonsillar cells for 1 or 3 h. We found that some transcripts were commonly expressed at both time points, while others were expressed uniquely at 1 or 3 h exposure to host cells. Specifically, at 3 h, we observed differentially expressed genes such as *sbnABCDE, isdACHF, dps, sle1, icaA*, and *scn_3*. Conversely, at the 1 h time point, we observed differentially expressed genes such as *metE, sdrD, msrA2, emp, chp, isdB* and *sbnH*. Notably, some genes such as *fnbA, fnbB*, and *isaA* were commonly downregulated at both time points. These findings indicate the transcriptional response of *S. aureus* during interaction with tonsillar cells, suggesting that these genes may play an important role in *S. aureus* throat colonization.

*S. aureus* exhibited almost 1 log10 difference in growth in presence of host cells compared to growth in absence of host cells. This observation suggests that the bacteria may be receiving additional nutrients from host, e.g., following host cell lysis, as indicated by the slight increase in LDH released by the infected host cells compared to the uninfected cells. The *sle1* gene (also known as *aaa*) is one of the important peptidoglycan hydrolases associated with cell separation in *S. aureus* [23, 24]. Interestingly, *sle1* was upregulated after 3 h exposure to host cells, perhaps as a consequence of the bacterial growth in presence of host cells.

*S. aureus* adhesion to host cells is a prerequisite for colonization and is therefore considered a major risk factor for subsequent development of staphylococcal infection [25]. A total of 35 *S. aureus* adhesins have been examined previously [6, 26, 27]. *S. aureus* surface proteins, including clumping factor B (ClfB), iron-regulated surface protein A (IsdA), serine-aspartate repeat-containing protein (Sdr)C, SdrD and surface protein G (SasG), as well as wall teichoic acid, have been identified to promote *S.aureus* adherence to nasal epithelial cells and is involved during nasal colonization [26, 28–31]. Notably, our transcriptomics data show nine significantly differentially expressed genes (*isdA*, *isdB*, *isdH*, *sdrC*, *sdrD*, *fnbA*, *fnbB*, *isaA* and *spa*) encoding surface-bound proteins. This is consistent with observations reported in previous studies indicating the differential expression of *sdrC, sdrD, isdA* and *IsdB* [32–34] in *S. aureus* in the presence of host.

Comparison of *in-vivo* and *in-vitro* gene expression profiles across different human niches has shown that *S. aureus* colonization of the anterior nares is strongly controlled by adhesins and iron availability [7, 35]. In this study, we found that most of the *S. aureus* iron regulated surface determinants (*isd*) genes were highly upregulated after 3 h of *S. aureus* exposure to tonsillar cell. This finding was consistent with a similar study performed during *S. aureus* vaginal colonization [21]. The Isd system modulates the acquisition of heme enabling the bacterial to extract nutrients such as iron from its environment [36]. Furthermore, we found that several other genes important in iron homeostasis such as *sbnA, sbnB, sbnC, sbnD, sbnE* and *sbnH* were also expressed. These genes are responsible for encoding proteins for the biosynthesis of staphyloferrin B (*sbn*ABCDEH) and its transport system (SirABC) [37].

*S. aureus* employs various mechanisms to evade host immune defences for its survival within the host [25, 38]. In our study, we found that *scn_3* (encoding Staphylococcal complement inhibitor) and *chp* (encoding Chemotaxis inhibitory protein), involved in countering the first line of host defence mechanisms were downregulated when *S. aureus* was exposed to host cells. This is contradictory to an earlier finding [7], which could potentially be attributed to the absence of neutrophils in our experimental setup. On the other hand, catalase (*katA*)was upregulated consistent with reports from a previous study [7]. Catalase play a crucial role in protecting cells against the toxic effects of hydrogen peroxide, and it is required for survival, persistence, and nasal colonization of *S. aureus* [39]. Similarly, the Dps family protein, which protects DNA under starvation conditions was also upregulated only after 3 h. This suggests that the expression of stress response genes may play a major role in the survival of *S. aureus* during throat colonization. The differential expression of these genes further indicates the dynamic adaptation of *S. aureus* to the host environment, particularly in response to the host immune defenses and nutrient availability.

The DEGs analysis of *S. aureus* cocultured with tonsillar cells, also revealed that various amino acid biosynthesis operons were upregulated. Notably, methionine synthase (*metE*) was reported to be upregulated only during 1 h of exposure, while *metC* was strongly upregulated at 1 h compared to 3 h. Previously, cystathionine-v-synthase (*metI*) has been reported to be strongly expressed during *S. aureus* colonization [30, 37, 40]. Upregulation of other methionine biosynthesis genes like cystathionine-b-lyase (*metC*), *metE, metH*, and *metI* including two L-methionine ABC-transport systems (*metN* and *metN2*) have also been reported [7]. These upregulated methionine biosynthesis genes represent a potential target for new antimicrobial strategies [41, 42] for combating *S. aureus* infection.

In our study, *fnbA* and *fnbB* were downregulated after both 1 and 3 h of exposure to tonsillar cells. This contrasts with previous studies indicating the role of FnbA and FnbB in promoting bacterial adhesion, biofilm formation and infections [43, 44]. Our findings suggest that these surface proteins [45] might be less relevant for *S. aureus* in presence of tonsillar cells in our experimental conditions. Transcription of *fnbA* and *fnbB* is downregulated in the post exponential phase of *S. aureus* growth [46]. This raises the possibility that the bacterial growth phase at the selected time points in this study may have influenced the expression pattern of *fnbA* and *fnbB*.

The common DEGs are important when *S. aureus* are exposed to a tonsillar cell line at both the time points, and the unique expression pattern might represent the importance of those genes either at 1 or 3 h. Overall, the transcriptome of *S. aureus* at 3 h showed increased upregulation of genes associated with iron acquisition while this was not observed at 1 h. This may suggest that over time *S. aureus* experiences a reduced level of available iron and increased competition for the ion [47, 48]. While *icaA* was upregulated at 3 h, potentially enhancing bacterial adhesion, the expression of *icaD*, a gene encoding IcaD that functions together with IcaA, was found to be commonly shared but with higher levels at 3 h compared to 1 h (lfc 5 at 1 h and lfc 6.5 at 3 h). Similarly, *sdrC* was noted as a commonly upregulated gene during the meeting of *S. aureus* with the tonsillar cell line. The level of expression of *sdrC* at 3 h (lfc 3.8) was higher than at 1 h (lfc 2.7), which may suggest increased SdrC-mediated adherence to tonsillar cells over time.

One of the limitations in our study, is that only one *S. aureus* strain and one type of mammalian cell line were included in the RNA-seq experiment. Another limitation is low mapping efficacy obtained in the 1 h test samples. To recover higher concentration of RNA required for RNA-seq, the in vitro experiment could have been performed in bigger cell culture dishes.

In conclusion, we have shown the suitability of using HTEpiC as an in vitro model for investigating key determinants in *S. aureus* involved in throat colonization. Our results reveal that some genes are commonly expressed, while others are uniquely expressed either at 1 or 3 h, indicating adaptation to the environment in presence of the tonsillar cell line. The up-regulated genes might be targets for intervention to prevent *S. aureus* throat colonization or infection in the future. Investigating the impact of other bacterial strain(s) present in the throat microbiome, as well as transcriptomic analysis of host cells using an ex-vivo model, could provide further insights into how *S. aureus* survival factors are influenced during co-colonization of the throat niche.

## Methods

### Experimental design

**Fig. 9:**
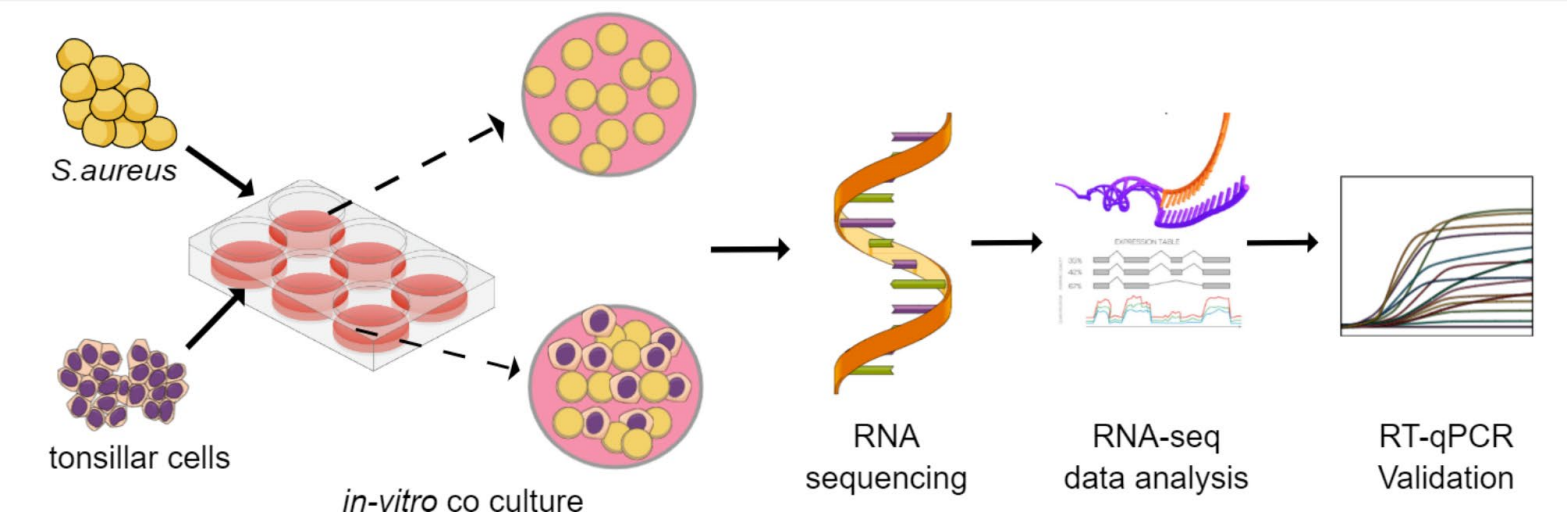
Overview of the experimental setup in this study

An overview of the experimental setup in this study is illustrated in Fig. 9. A Staphylococcus aureus throat isolate was cultured with or without a tonsillar cell line prior to RNA-seq to find differentially expressed genes (DEGs).

### Human tonsil epithelial cells

Human Tonsil Epithelial Cells (HTEpiC) were purchased from Sciencell, United States (Cat #2560) and was isolated from a 10-year-old male. The tonsillar cells were cultured using Tonsil Epithelial Cell Medium (TEpiCM, Sciencell, Cat #2561) supplemented with 1% Tonsil Epithelial Cell Growth Supplement (TEpiCGS, Sciencell, Cat #2572) and penicillin/streptomycin solution (P/S, Sciencell, Cat #0503) (hereafter referred to as complete medium) at 37 °C in a 5% CO_2_ incubator. Prior to the culturing of HTEpiC, the T-75 tissue culture flask was coated with 2 µg/cm^2^ poly-L-lysin (PLL) (Sciencell, Cat #0403) and incubated at 37 °C for 2 h or overnight. Morphology, growth, and multiplication of HTEpiC were checked regularly. The complete medium was changed every third day until the cells reached 70% confluency and thereafter every second day until 90% confluent culture was observed (Additional file 6, Figure S2). At approximately 90% confluency, subculturing was initiated with trypsinization of cells using 0.25% trypsin/EDTA solution (T/E, Cat #0183) and handled according to the manufacturer´s instructions (Sciencell Research Laboratories, California).

### Bacterial strains and growth conditions

A *Staphylococcus aureus* strain TR145 (spa-type t045, clonal complex 15) isolated from the throat in a healthy, adult individual in the Tromsø 6 study [49, 50] was used in this study. A single colony of *S. aureus* TR145 was transferred to 10 ml of Tryptic Soy Broth (TSB) and the culture was incubated overnight with shaking at 220 revolutions per minute (rpm) at 37 °C. One ml of overnight *S. aureus* culture was inoculated into 9 ml fresh TSB and incubated at 37 °C with shaking at 220 rpm for 1–2 h to reach the logarithmic growth phase (0D_600_ 0.8–1.2). Freshly prepared logarithmic growth culture was subsequently harvested by centrifugation at 5000 rpm for 10 min at room temperature. The bacterial pellet was washed twice with autoclaved phosphate-buffered saline (PBS) and dissolved in 1 ml PBS.

The bacterial culture was adjusted to OD = 0.4 (corresponding to approximately 1 × 10^8^ CFU/ml) and used as inoculum to infect the host cells. To confirm the colony forming units (CFU), serial dilution of the inoculum was done followed by plate enumeration. The plating was done in triplicate onto TSA plates and left to incubate for 24 h at 37 °C. The bacterial colonies were then counted, and the average CFU/ml was calculated.

### In vitro culturing of ***S. aureus*** with/without tonsillar cell line

**Fig. 10:**
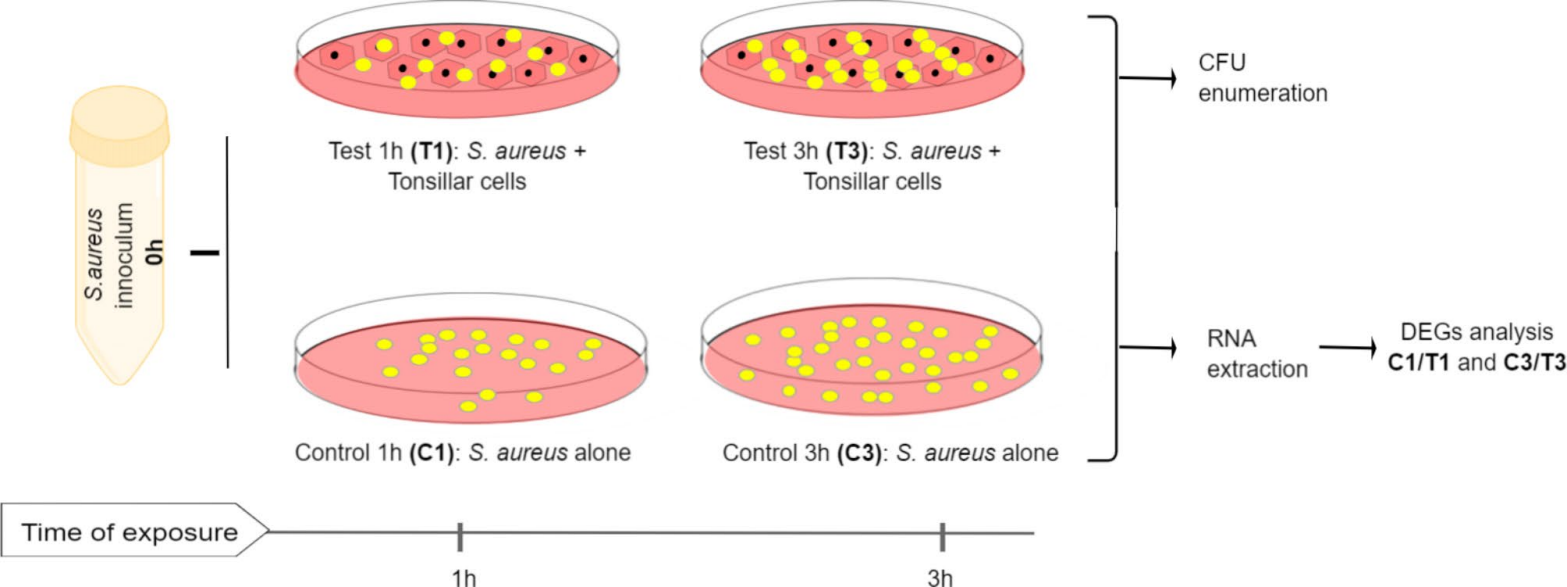
Schematic representation of the in vitro co-culturing of *S. aureus* with or without tonsillar cells. *S. aureus* (inoculum) was added to PLL-coated wells containing host media in absence (controls) or presence of monolayer of host cells (tests) at MOI 5 and incubated for 1 or 3 h. Three independent experiments were run in triplicates. The adhered bacteria were collected and plated for either CFU enumeration or RNA extraction. The RNA samples were further processed for RNA-seq followed by DEGs analysis (C1/T1 and C3/T3)

HTEpiC was cultured until passage four and seeded at a density of ~ 4 × 10^5^ viable cells per well in six well plates for adhesion assay or ~ 7 × 10^4^ viable cells per well in 24 well plates coated with PLL. The HTEpiC was grown until confluence, washed with Dulbecco’s Phosphate-Buffered Saline (DPBS, Sciencell, Cat #SC0303) and added TEpiCM. *S. aureus* TR145 at OD_600nm_ of 0.4 in complete medium (without antibiotics) was added to HTEpiC monolayer or to empty wells in a number corresponding to Multiplicity of Infection (MOI) = 5 and incubated for 1 and 3 h (Fig. 10, to capture the initial stages of *S. aureu*s-host cell interaction [51, 52]. Images of host cells in absence or in presence of *S. aureus* for 1 and 3 h are shown in Additional file 6, Figure S3.

After 1 and 3 h post-infection, the media was aspirated, and the host cells were washed twice with fresh media/DPBS to remove unbound bacteria. The host cells were then trypsinized and lysed with Triton-X. The released bacteria were then collected from three technical replicates and pooled together. At both time points, bacteria seeded into PLL coated plates without host cells (Fig. 10, control 1 h and control 3 h) were also collected using scraping technique followed by visual inspection of the wells by microscopy to ensure that most of bacteria were recovered from the well.

An aliquot of the bacterial suspensions was serially diluted and plated on TSA agars for CFU determination. The remaining bacteria were centrifuged immediately at 5000 rpm at room temperature for 10 min. The bacterial pellets were resuspended in 100 µl of RNAprotect® Bacterial Reagent (Qiagen, Cat #76,506) followed by 5 s vortexing and incubation for 5 min at RT. After the final centrifugation (5000 rpm, 10 min, room temperature), bacterial pellets were preserved at -80 °C until total RNA isolation.

### Cytotoxicity assay

Host cell lactate dehydrogenase (LDH) released into supernatants after 1 and 3 h of post-infection was quantified using CytoTox96®Non-Radioactive Cytotoxicity Assay (Promega, G1781), according to the manufacturer’s instructions. Positive control (HTEpiC infected with *S. aureus*) included in the cytotoxicity assay represented 100% cell death after adding 2 µl of lysis solution (9% W/V Triton-X-100) per 100 µl volume. Both background control (only complete medium) and negative control (non-infected HTEpiC) were included for each condition at both time points. For the quantification of the sample, colorimetric measurement of LDH release was measured at 490 nm using a standard 96-well plate reader, analyzed using SoftMax Pro Software. Sample readings (from three technical replicates) were divided by the positive control for cell lysis to result in a percentage of total cell death for each sample.

### Bacterial lysis and RNA preparation

Bacterial pellets in RNAprotect® at -80 °C were thawed and suspended in 100 µl of TE buffer (10 mM Tris Cl, 1 mM EDTA, pH 8; Sigma-Aldrich) containing the lysozyme (0.5 µl, 0.1 mg/ml; Sigma-Aldrich) and lysostaphin (0.5 µl, 10 mg/ml; Sigma-Aldrich) and incubated at 37 °C for 10 min. The bacterial suspensions were transferred to a 0.5 ml Safe-Lock centrifuge tube containing acid-washed glass beads (0.1 mm diameter, Cat.No. 11,079,101, BioSpec product) and disrupted using *Precellys® Evolution* homogenizer (Precellys Evolution, bertin technologies) at 4500 rpm, 40 s x 2 cycle, 4 min pause on ice.

After homogenization, total RNA was isolated, following the recommendations of the manufacturer (Qiagen RNeasy Mini Kit, Cat.No. 74,104). RNA was eluted with 40 µl of nuclease-free water (Ambion; Darmstadt, Germany) and the eluate was used to re-eluate (30–35 µl) to achieve higher RNA concentration. DNase treatment was performed using Heat and Run kit (ArcticZymes, Norway) according to the manufacturer’s instructions. RNA quantity and integrity were measured by Nanodrop1000 spectrophotometer (Thermo Scientific; Waltham, MA, USA or Biolab), and Agilent 2100 Bioanalyzer (Agilent Technologies, Santa Clara, CA, USA), respectively.

### NGS library construction and RNA-sequencing

Total RNA extracted from three replicates of *S. aureus* TR145 grown in absence of host cells collected at time points of 1 and 3 h (*S. aureus* only, control samples (C)) and three replicates of *S. aureus* TR145 after 1 and 3 h exposure to host cells (*S. aureus* + tonsillar cells, test samples (T)) were selected for RNA-seq library preparation (Table 3).

Depletion of rRNA was performed with the RiboCop depletion kit (Lexogen, cat no: 127 (RiboCop rRNA depletion kit for Gram Positive Bacteria (G+)), according to the manufacturer’s protocol. In total, 12 RNA samples (Table 3) were processed for library construction using Lexogen’s CORALL™ Total RNA-Seq Kit with RiboCop (Cat.No.96; EU, CH, USA).

**Table 3:** Twelve RNA samples were processed for NGS library preparation and RNA-seq

Replicate number	Sample ID	Group
1st Biological replicates	*S. aureus* only _1h	C1h_Rep1
	*S. aureus* only _3h	C3h_Rep1
	*S. aureus* + tonsillar cells_1h	T1h_Rep1
	*S. aureus* + tonsillar cells_3h	T3h_Rep1
2nd Biological replicates	*S. aureus* only _1h	C1h_Rep2*
	*S. aureus* only _3h	C3h_Rep2
	*S. aureus* + tonsillar cells_1h	T1h_Rep2
	S. aureus + tonsillar cells_3h	T3h_Rep2
3rd Biological replicates	*S. aureus* only _1h	C1h_Rep3
	*S. aureus* only _3h	C3h_Rep3
	*S. aureus* + tonsillar cells_1h	T1h_Rep3
	*S. aureus* + tonsillar cells_3h	T3h_Rep3

Note *This control sample had the lowest RNA concentration and did not reveal the presence of RNA after library preparation and was therefore not processed for RNA-seq library preparation

All samples were run with 16 PCR cycles for the final library amplification step. One of the samples had too low concentration (~ 1.5 ng/µl) after fragmentation and did not proceed for sequencing. Otherwise, all steps were according to the manufacturer’s protocol. The samples were sequenced on an Illumina 550 platform, with dual indexes, and paired end mode. The final sequencing concentration was 1.8 pM.

### RNA-seq data analysis

**Fig. 11:**
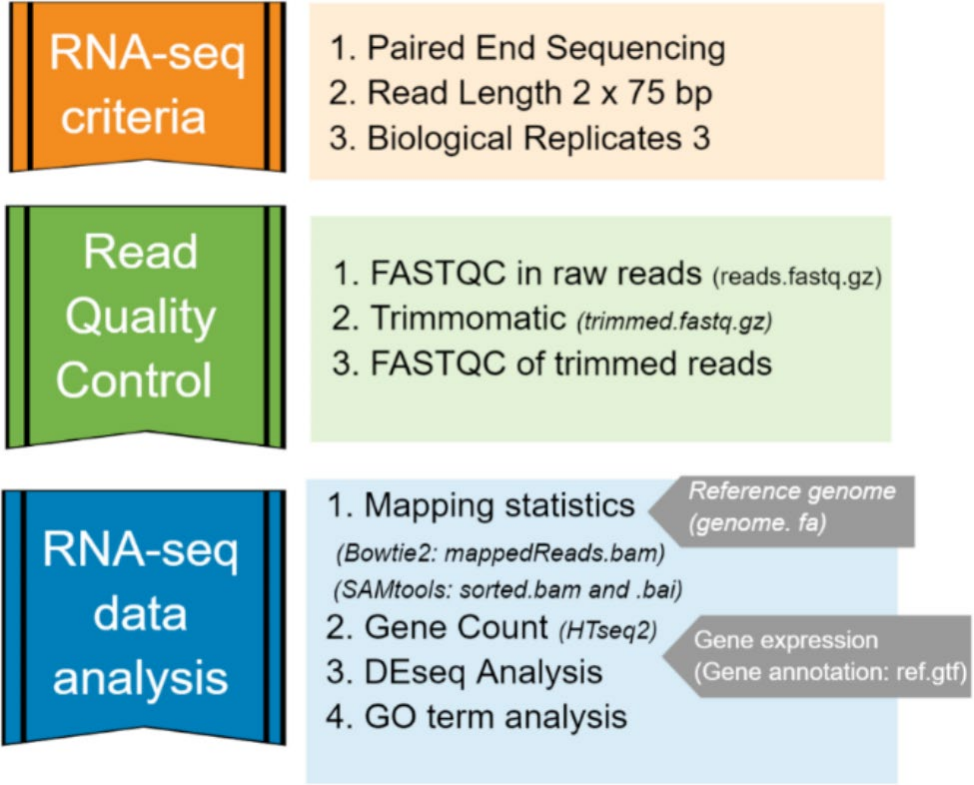
RNA-seq workflow analysis used in this study

Total RNA reads were generated from 11 samples in two runs of RNA-seq (Table 3). The illumina paired end data were then mapped against *S. aureus* TR145 reference genome to retrieve bacterial reads that were uniquely mapped. The remaining most reads representing eukaryotic RNA were eliminated bioinformatically. Only bacterial reads were further processed for differentially expressed genes (DEGs) analysis. The detailed RNA-seq bioinformatics pipeline is illustrated in Fig. 11.

Each library was pre-processed for quality check using FASTQC/0.11.9-Java-11 (https://www.bioinformatics.babraham.ac.uk/projects/fastqc/*).* Filtering (removable of adaptor dimer reads) and trimming (removable of low-quality bases) was performed by Trimmomatic/0.39-Java-11 (http://www.usadellab.org/cms/?page=trimmomatic*).* Only those sequences with quality score Q > 20 and a minimum of 55 nucleotide sequence length were retained in the dataset. The final quality check was performed in the trimmed file. *S. aureus* strain TR145 (SAMEA112465883) was previously whole genome sequenced and kindly provided to us (M Røkeberg Olsen, unpublished results). The genome from *S. aureus* TR145 was used as a reference genome for the mapping performed using Bowtie2/2.4.4-GCC-10.3.0 (https://bowtie-bio.sourceforge.net/bowtie2/index.shtml*).*

Reads mapped to reference (*S. aureus* TR145) gene was identified using HTSeq counting tool (https://htseq.readthedocs.io/en/release_0.11.1/count.html). After aligning reads to a reference and generating count files, it was further analyzed by DESeq2 (https://bioconductor.org/packages/release/bioc/html/DESeq2.html) to explore any DEGs present in the sample. The DE was further curated to give only those genes which show p- value adjusted (padj) along the indication of gene name. Threshold for padj was adjusted to less than 0.05 (padj < 0.05) and for log2fold change greater than 2 (lfc ≥ |2|). Any gene that followed these two thresholds was a good starting point for identifying significant genes. The DEGs were visualized from plots such as PCA and Venn diagram in R using ggplot2. Further, these DEGs were analyzed using ShinyGO 0.76.2 (http://bioinformatics.sdstate.edu/go/), a graphical tool for gene ontology (GO) enrichment analysis. DEGs being involved in different GO terms such as molecular function, biological process, and cellular components were identified. The GO terms with false discovery rate (FDR) less than 0.05 were considered significantly enriched.

### Validation by qRT-PCR

Validation of RNA-seq data was performed by qRT-PCR. Six genes with different expression profiles were selected based on fold change, p-value, and functions. For instance, gene with highest fold change and lowest p-value (*ilvC*), gene with medium fold change but commonly expressed (*metI*), uniquely expressed genes involved in cellular process (*metI, emp*, *icaA*), molecular function (*rspt*), and both in the molecular function and biological process (*metE*) (Table 4). The mRNA transcripts of the selected DEGs were quantified using the LightCycler® 96 Instrument Software, Version 1.1.1 - Service Pack SP1 according to the manufacturer’s instructions (Table 4).

**Table 4:** Primer sequence and expected amplicon size of the six selected genes used for qRT-PCR in this study

Gene	Primer Sequence (5’ -3’)	Amplicon size (bp)	Efficiency E (%)	Correlation coefficient (R^2^)
*groEL*	GCACCAGTTCGTCAAATTGC (L) CACTCGTTTGTAGCAGCGTT (R)	110	115	1.00
*ilvC*	AAACGGACGCTTTACAAGGC (L) GTCAAAAGAACGACCTGGGC (R)	131	121	0.97
*metI*	ACATGGTATTGCATCATTCGCT (L) ATGTGCCGCCGTATAAATCG (R)	116	120	0.94
*metE*	CGAAAGCGTGCGTACTTCAA (L) TCTCGGCTTTGTGGGAATGA (R)	120	118	0.90
*icaA*	CGACGTTGGCTACTGGGATA (L) TGCTTCCAAAGACCTCCCAA (R)	150	122	0.94
*emp*	CGCGTGAATGTAACAACAAACA (L) CTTGTAGTGGGTTTGCGTAGT (R)	138	114	0.98
*rpsT*	CTGAAGCACGCAACATTTCAC (L) ACTTTGAGCAGCTTTGTCTACT (R)	140	119	0.92

RNA was reverse transcribed into cDNA using the High-capacity cDNA Reverse Transcription kits (Applied Biosystems, Forster City, CA, USA, Cat #4,368,814) according to the manufacturer’s protocol. Two microliter of generated cDNA / negative RT control was used as a template for the generation of amplicons from gene of interest. Each qRT-PCR reaction was performed in a final volume of 25 µl. The final concentration of 100 nM of each primer pair was added to 12.5 µl of SYBR® Green PCR Master Mix (1X, Takyon™ qPCR MasterMixes for SYBR® assays containing Low ROX passive reference, Eurogentec, Fremont, CA, USA, Cat #UF-LSMT-B0701). The PCR reactions were generated in a thermal cycler: 95 °C for 15 s; 40 cycles of 60 °C for 60 s, 95 °C for 15 s, and a final extension of 60 °C for 15 s. RT negative control, and PCR Negative control samples with sterile water were also included. All qRT-PCR experiments were performed using three biological replicates and three technical replicates. The Quantification cycle values (Cq) were determined, and relative fold differences were calculated by Delta-Delta Cq method using *groEL* as the reference gene. The expression profiles of the six genes analyzed relative to the *groEL* was compared with the values of Deseq2 analysis obtained from the RNA-Seq data.

## Electronic supplementary material

Below is the link to the electronic supplementary material.

Supplementary Material 1

Supplementary Material 2

Supplementary Material 3

Supplementary Material 4

Supplementary Material 5

Supplementary Material 6

## Data Availability

Raw RNA-seq reads and processed files generated in this study can be found in the European Nucleotide Archive (ENA) repository with the GEO accession number GSE226317 (https://www.ncbi.nlm.nih.gov/geo/query/acc.cgi?acc=GSE226317) under the project number PRJNA939634. The FASTQ files of reference genome (*S. aureus* TR145 strain) and annotation files used in this study is deposited in ENA with BioSample accession SAMEA112465883 (https://www.ebi.ac.uk/ena/browser/view/PRJEB59355) under the project number PRJEB59355. The datasets used and/or analyzed during the current study are available from the corresponding author on reasonable request.

## Abbreviations

*aldh1*: Aldehyde dehydrogenase 1
*Clf*: Clumping factor
cDNA: Complementary Deoxyribonucleic acid
CFU: Colony forming units
chp: Calcineurin B homologous protein
cna: Collagen adhesion
cq: Quantification cycle
DEGs: Differentially expressed genes
dps: DNA protection during starvation protein
DPBS: Dulbecco’s Phosphate-Buffered Saline
eap: Extracellular adherence protein
ebh: Extracellular matrix-binding protein ebh
*ebps*: Elastin-binding protein EbpS
*ecm*: Extracellular matrix
*emp*: Extracellular matrix protein-binding protein
FnBPs: Fibronectin binding proteins
HaCaT: Human Keratinocyte Cell Culture
HTEpiC: Human tonsil epithelial cells
*icaA*: Intracellular adhesion A
*ilvC*: Ketol-acid reductoisomerase
GO: Gene ontology
*isaA*: Immunodominant staphylococcal antigen A
isd: Iron-regulated surface determinants
katA: Catalase
*leuC*: Leucine biosynthesis
LDH: Lactate dehydrogenase
*met*: Methionine biosynthesis
MRSA: Methicillin-resistant *Staphylococcus aureus*
MSSA: Methicillin-sensitive *Staphylococcus aureus*
MSCRAMMs: Microbial surface components recognizing adhesive matrix molecules
MOI: Multiplicity of infection
NEAT: NEAr Transporter domain
0D_600_: Optical density
PCA: Principal component analysis
PBS: Phosphate-buffered saline
P/S: Penicillin/streptomycin solution
PLL: Poly-L-lysin
qRT-PCR: Quantitative reverse transcription polymerase chain reaction
*rpsT*: Encoding ribosomal protein S20
RNA-seq: RNA-sequencing
rpm: Revolutions per minute
*sas*: Staphylococcus aureus surface protein
*Scn_3*: Staphylococcal complement inhibitor 3
*sbn*: Staphyloferrin B biosynthesis protein
*sdrC*: Serine-aspartate repeat-containing protein C
*sle1*: Cell separation gene of *S. aureus*
*spa*: Staphylococcal protein A
*sraP*: Serine-rich adhesion for platelets
SNM: Synthetic nasal medium
TEpiCM: Tonsil Epithelial Cell Medium
TEpiCGS, Tonsil Epithelial Cell Growth Supplement; T/E: Trypsin/EDTA solution
TSB: Tryptic soy broth
