# Supplementary material for: Exploring differentially expressed genes of Staphylococcus aureus exposed to human tonsillar cells using RNA sequencing
Source: BMC Microbiol. 2023 Jul 12;23:185. doi: 10.1186/s12866-023-02919-5 (PMC10337072; doi:10.1186/s12866-023-02919-5)
Supplement: Supplementary file 6 — Supplementary Material 6 [file 12866_2023_2919_MOESM6_ESM.pdf]

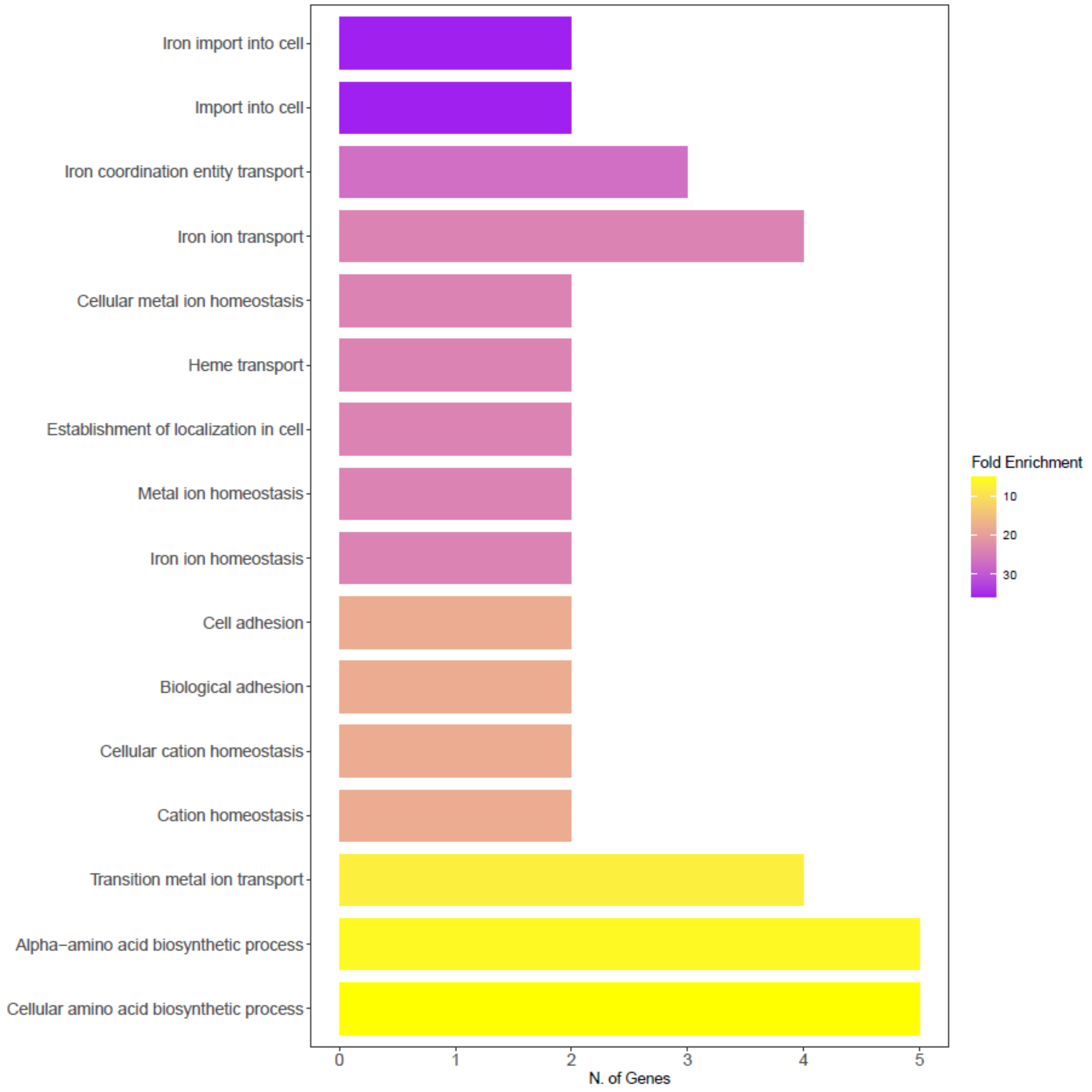

***Figure S1: Top 16 significantly enriched GO terms involved in biological process analyzed from differentially expressed genes uniquely upregulated at 3h of exposure to host cells**. Genes were significantly involved (FDR < 0.05) in the respective pathways detected by GO enrichment analysis.*

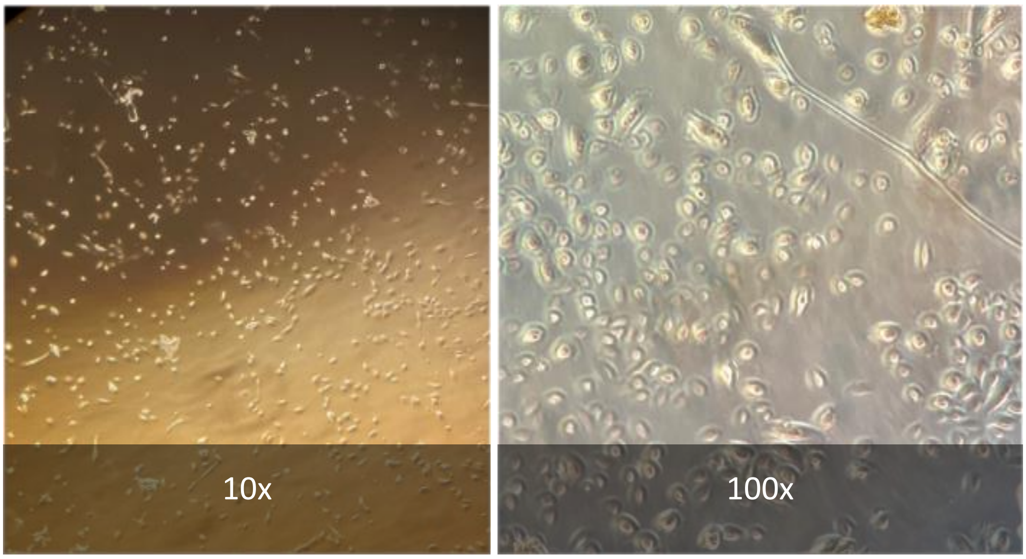

Figure S2: Microscopic view of Human Tonsil Epithelial Cells (HTEpiC) at 10x and 100x magnifications. *Images shows healthy and dividing HTEpiC at passage 4, which is ready to be infected.*

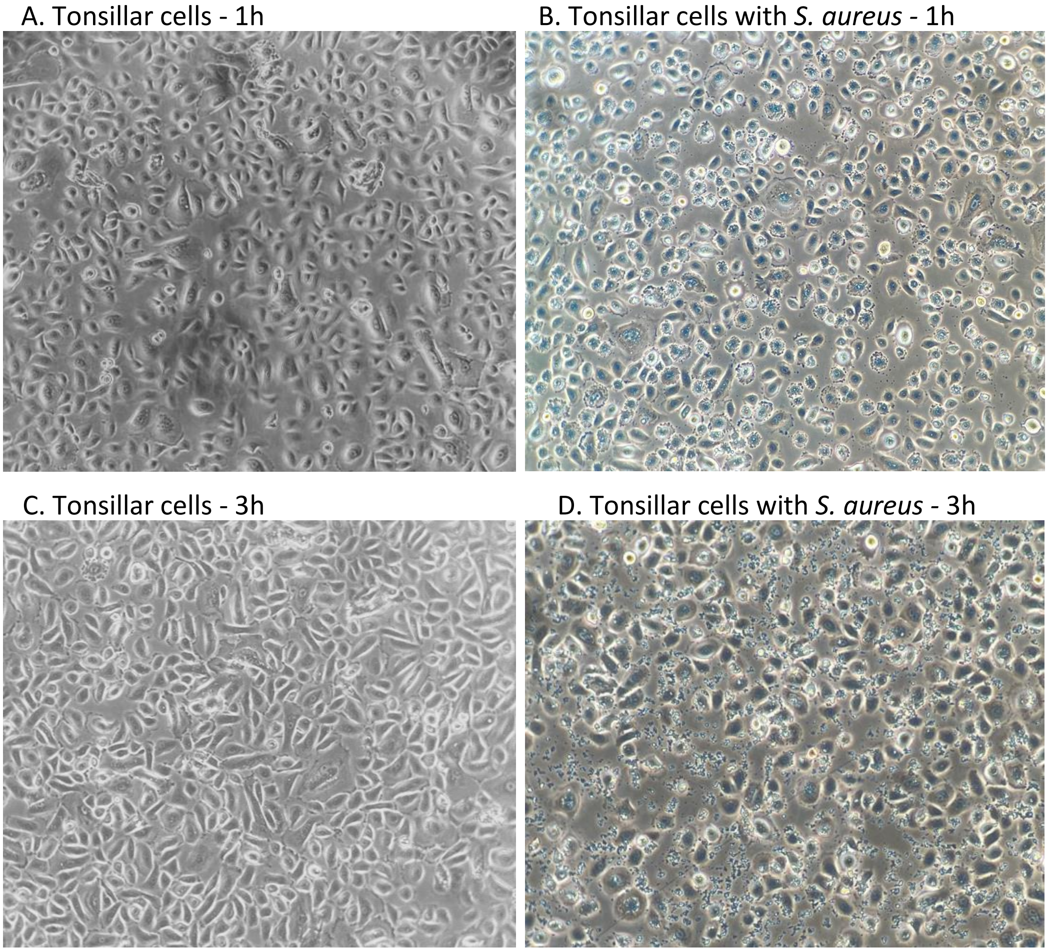

Figure S3: Microscopic view of Human Tonsil Epithelial Cells (HTEpiC) in the absence or presence of S. aureus. A) *Tonsillar cells incubated for 1h in the absence of S. aureus.* ***B)*** *Tonsillar cells incubated for 1h of in the presence of S. aureus.* ***C)*** *Tonsillar cells incubated for 3h in the absence of S. aureus.* ***D)*** *Tonsillar cells incubated for 3h with S. aureus. Bacteria are seen as dots or aggregates in B and D.*
